# Isoforms of cysteine peptidases and osmotins instead of chitinases and peptidase inhibitors drive antifungal activity but occur as distinct isoforms in latex and callus tissues of *Calotropis procera*

**DOI:** 10.1007/s00425-026-05198-2

**Published:** 2026-10-11

**Authors:** Márcio V. Ramos, Rayanne F. Silva, Cristina P. S. Carvalho, Arlete A. Soares, José Francisco C. Gonçalves, Patrícia M. S. Carvalho, Pedro A. V. R. Júnior, João M. Matos-Neto, Cleverson D. T. Freitas

**Affiliations:** 1 Departamento de Bioquímica E Biologia Molecular, Centro de Ciências, Universidade Federal Do Ceará, Bloco 907, Campus Do Pici, Fortaleza, Ceará CEP 60440-554 Brazil; 2 Department of Biology, Federal University of Ceará, Fortaleza, Ceará CEP 60440-554 Brazil; 3 Laboratory of Plant Physiology and Biochemistry, National Institute for Amazonian Research - INPA, Manaus, Amazonas CEP 69011-970 Brazil

**Keywords:** *Cryptostegia grandiflora*, Pathogenesis-related proteins, Plant defense mechanisms, Tissue-specific gene expression

## Abstract

**Main conclusion:**

Peptidases and osmotins are the primary determinants of antifungal activity, and their expression is tightly linked to tissue specialization in *Calotropis procera* plants.

**Abstract:**

This study investigated whether callus cultures of two Apocynaceae species, *Calotropis procera* and *Cryptostegia grandiflora*, reproduce the antifungal defense system typically found in their latex. Protein fractions obtained from latex and callus tissues were evaluated for antifungal activity against *Fusarium solani*, *F. oxysporum*, *Colletotrichum lindemuthianum*, and *C. gloeosporioides*, as well as the presence of pathogenesis-related (PR) proteins was investigated. Callus proteins from *C. procera* (CPCp) exhibited antifungal activity, inhibiting both mycelial growth and spore germination of all tested fungi. In contrast, callus proteins from *C. grandiflora* (CPCg) showed no detectable antifungal activity. Mechanistic assays demonstrated that CPCp induced plasma membrane permeabilization and the accumulation of reactive oxygen species in fungal spores. Biochemical analyses further revealed that antifungal activity was associated with the presence of peptidases and osmotins but not of peptidase inhibitors and chitinases. Immunological recognition and gene expression analyses indicated that the peptidases and osmotins expressed in callus tissues differ from those present in the latex of *C. procera*, suggesting the occurrence of distinct protein isoforms. Collectively, these findings demonstrate that peptidases and osmotins are key determinants of antifungal activity in latex plants and reveal that their expression is closely linked to tissue specialization, highlighting the differential regulation of defense protein isoforms between latex and other plant tissues.

**Supplementary Information:**

The online version contains supplementary material available at https://doi.org/10.1007/s00425-026-05198-2.

## Introduction

Laticifers are specialized secretory structures that produce latex, a complex mixture of metabolites and proteins that is rapidly released upon tissue damage (Pickard 2008). Latex is widely distributed among vascular plants and occurs in approximately 6% of plant species, being particularly abundant in families such as Apocynaceae, Euphorbiaceae, Moraceae, and Papaveraceae (Hagel et al. 2008). This secretion contains a diverse array of bioactive compounds, including alkaloids, terpenoids, and numerous proteins associated with plant defense (Agrawal and Konno 2009). The accumulation of these molecules at high concentrations within laticifer cells has led to the hypothesis that latex plays an important role as a defensive barrier against herbivores and microbial pathogens, acting both as a physical sealant and as a reservoir of toxic or antimicrobial compounds (Ramos et al. 2019).

Several classes of pathogenesis-related (PR) proteins have been reported in latex, including peptidases, chitinases, β-1,3-glucanases, osmotins, and peptidase inhibitors (Ramos et al. 2020). These proteins contribute to plant defense through different mechanisms, such as degradation of fungal cell wall components, disruption of plasma membrane integrity, inhibition of digestive enzymes in herbivores, or induction of oxidative stress in invading pathogens (Freitas et al. 2024). In particular, cysteine peptidases represent one of the most abundant enzymatic classes in latex and have been widely associated with antifungal activity (Barbosa et al. 2020). Likewise, osmotins, members of the PR-5 protein family, inhibit fungal growth through mechanisms involving membrane permeabilization, oxidative stress, and induction of programmed cell death (Ullah et al. 2018).

Despite the extensive characterization of defense proteins in latex, it remains unclear whether genes encoding these proteins are expressed beyond laticiferous tissues and whether the corresponding protein forms are conserved across tissues. Comparative analyses of gene expression and protein profiles in laticiferous and non-laticiferous tissues may provide insights into the tissue specificity of these defense components. Therefore, in vitro plant tissue culture systems provide a valuable experimental model to address this question because they allow the generation of undifferentiated tissues, such as callus, which lack specialized secretory structures like laticifers (Malabadi et al. 2025). If the proteins typically found in latex are constitutively expressed throughout the plant, they should also be detected in callus cultures. Conversely, if their biosynthesis depends on laticifer differentiation, these proteins may be absent or replaced by alternative isoforms in non-laticifer tissues. However, this hypothesis has rarely been experimentally tested in laticiferous plants. Teixeira et al. (2011) provided an initial approach to this question in *C. procera* by investigating callus cultures devoid of laticifer differentiation and assessing whether they retained pharmacological activities previously attributed to latex proteins. Although callus-derived proteins exhibited some activities also reported for latex, the distinct protein profiles observed between the two tissues suggested that callus cultures produce a different repertoire of bioactive proteins. Nevertheless, the proteins underlying these activities were not characterized, and it therefore remained unclear whether the bioactive proteins produced by callus correspond to those accumulated in latex or represent distinct protein isoforms.

In this context, the present study investigated whether callus cultures of two laticiferous species, *C. procera* and *C. grandiflora*, somehow reproduce the antifungal defense system previously described in their latex. By combining histological analyses, antifungal assays, biochemical characterization, immunological detection, and PCR analysis of some PR-proteins, we evaluated the occurrence of key defense proteins and their possible mechanisms of action against phytopathogenic fungi. Our results demonstrate that antifungal activity is associated with the presence of peptidases and osmotin-related proteins. Furthermore, we show that the peptidases and osmotins expressed in callus tissues differ from those present in latex, indicating the occurrence of tissue-specific protein isoforms. These findings provide new insights into the functional specialization of laticifer defense systems and reveal that the expression of key antifungal proteins in latex plants is tightly linked to tissue differentiation.

## Materials and methods

### Biological material

*C. grandiflora* and *C. procera* were taxonomically identified by a specialist from the Prisco Bezerra Herbarium, Federal University of Ceará (UFC), where voucher specimens were deposited under accession numbers 040409 and 32,663, respectively. Healthy non-cultivated plants grown in Fortaleza, Ceará, Brazil, were used as sources of fresh latex and mature seeds. Seeds were manually removed from fruits, cleaned, and stored at 25 ± 2 °C until use.

The phytopathogenic fungi *Fusarium solani*, *F. oxysporum*, *Colletotrichum lindemuthianum*, and *C. gloeosporioides* were kindly provided by the Laboratory of Plant Defense Proteins, Department of Biochemistry and Molecular Biology, Federal University of Ceará, Brazil.

### Callus induction

Seeds (*n *= 25) of *C. grandiflora* and *C. procera* were surface-sterilized in 70% (v/v) ethanol for 5 min, followed by 1% (v/v) sodium hypochlorite for 20 min, and rinsed five times with sterile distilled water. *C. grandiflora* seeds were germinated on autoclaved sand under a 16 h photoperiod (fluorescent light at 10–15 µmol m^−2^ s^−1^) at 25 ± 2 °C. After four weeks, seedlings (*n *= 25) were used as explant sources. *C. procera* seeds (*n *= 25) were germinated on half-strength Murashige and Skoog (MS) medium supplemented with 2% (w/v) sucrose and 0.7% (w/v) agar, as previously described by Teixeira et al. (2011).

Hypocotyl (10 mm) and cotyledon segments (approximately 1 cm^2^) from both species (*n *= 20) were cultured on half-strength MS medium (pH 5.8) containing 2% sucrose, 0.7% agar, 3 μM 1-naphthaleneacetic acid (NAA), and 4.6 μM kinetin (KIN) for callus induction (Teixeira et al. 2011). Cultures were maintained in complete darkness at 25 ± 2 °C. After four weeks, calli were harvested for histological, biochemical, and biological analyses.

### Histological analysis

Histological analyses were performed exclusively on *C. grandiflora* samples, since these analyses have previously been reported for *C. procera* (Teixeira et al. 2011). Cotyledons, hypocotyls, and calli (*n *= 5) were fixed in Karnovsky’s solution (1% paraformaldehyde and 3% glutaraldehyde in 70 mM cacodylate buffer, pH 7.2) for 48 h (Karnovsky 1965). Samples (*n *= 5) were dehydrated through a graded ethanol series (10–100%, 1 h each), infiltrated with a 1:1 ethanol:resin mixture (Leica Historesin Embedding Kit) for 24 h, and sectioned at 5 μm using a Leica 2065 microtome. Sections were stained with Astra blue and safranin (9:1, v/v) (Roeser 1972) or with 0.12% toluidine blue in 5% borax, briefly counterstained with 0.05% basic fuchsin for 5 s, mounted with Entellan®, and examined under a Leica DM4000 B LED microscope.

### Protein extraction from calli and latex

Calli (*n *= 20) from both species were homogenized in 500 mM NaCl containing 10 mM ascorbic acid at a ratio of 1:2.5 (w/v). Homogenates were stirred for 90 min at 4 °C and centrifuged at 10,000 g for 10 min. Proteins were precipitated with ammonium sulfate (0–90% saturation) and extensively dialyzed against distilled water for 48 h using a dialysis membrane with a molecular weight cut-off of 14 kDa (Sigma-Aldrich, D9527) to remove residual salts and other low-molecular-weight compounds. The dialyzed protein fractions were then lyophilized and stored at 25 °C. Callus protein (CP) fractions were designated CPCg and CPCp for *C. grandiflora* and *C. procera*, respectively.

Fresh latex from both species was collected directly into distilled water (1:1, v/v) and centrifuged at 10,000 g for 10 min at 4 °C to remove rubber particles. Supernatants were dialyzed against distilled water for 48 h using a dialysis membrane with a molecular weight cut-off of 14 kDa (Sigma-Aldrich, D9527), centrifuged again, and lyophilized. Latex protein (LP) fractions were designated LPCg and LPCp for *C. grandiflora* and *C. procera*, respectively. Protein concentrations were determined using the Bradford method with bovine serum albumin as the standard (Bradford 1976).

### Antifungal activity

Antifungal activity against *F. solani*, *F. oxysporum*, *C. lindemuthianum*, and *C. gloeosporioides* was evaluated as described by Souza et al. (2011). Fungi were maintained on Sabouraud Dextrose Agar at 27 ± 2 °C in the dark and subcultured monthly. Spore suspensions were prepared by adding 5 mL of sterile distilled water to 15-day-old cultures, gently scraping the colony surface with a Drigalski loop, filtering through sterile nylon mesh, and counting spores using a Neubauer chamber. Suspensions were adjusted to 2 × 10^5^ spores/mL.

For spore germination assays, 10 μL of spore suspension were mixed with 10 μL of protein samples (5 mg/mL) and incubated at 27 °C in the dark (Souza et al. 2011). Germination was assessed microscopically after 24 h. For mycelial growth inhibition assays, 10 μL of spore suspension were incubated with 90 μL of yeast peptone dextrose medium for 16 h at 27 °C in 96-well plates, followed by the addition of 100 μL of protein samples (5 mg/mL). Sodium acetate buffer (50 mM, pH 5.0) and 1 mM H₂O₂ were used as negative and positive controls, respectively. Growth was monitored by measuring turbidity at 620 nm for 48 h using a Biotrak II microplate reader (Amersham Biosciences).

### Mechanism of action

Membrane integrity was evaluated using propidium iodide staining (Ramos et al. 2014). Spore suspensions (25 μL, 20 × 10^5^ spores/mL) were incubated with 25 μL of protein samples (5 mg/mL) for 30 min, followed by the addition of 10 μL of propidium iodide (1 mM). Samples were washed with sterile 0.15 M NaCl and examined under a fluorescence microscope (Olympus BX system). Sodium acetate buffer (pH 5.0) and 100 μM cetrimide were used as negative and positive controls, respectively.

Reactive oxygen species (ROS) production was assessed using 3,3′-diaminobenzidine (DAB), as previously described (Souza et al. 2011). Spores (25 μL, 20 × 10^5^ spores/mL) were incubated with protein samples (25 μL, 5 mg/mL) for 30 min, followed by the addition of DAB (0.1 mg/mL) and further incubation for 2 h at 27 °C. Samples were examined by light microscopy.

## Detection of plant defense proteins

### Cysteine and serine peptidase inhibitors

Cysteine peptidase inhibitor activity was evaluated using a papain inhibition assay, as previously described (Ramos et al. 2010). Protein extracts (100 µL, 5 mg/mL) were incubated with 15 µL of papain (0.1 mg/mL) for 30 min, followed by the addition of 200 µL of 1 mM Nα-benzoyl-DL-arginine-β-naphthylamide (BANA) as substrate. After 30 min, the reaction was stopped by adding 500 µL of 2% HCl in ethanol followed by 500 µL of 0.06% 4- (dimethyl-amino) cinnamaldehyde. After 40 min, the resulting color was measured by absorbance at 540 nm. Controls included protein extracts alone, papain incubated with E−64 (a specific cysteine peptidase inhibitor), and papain alone.

Serine peptidase inhibitor activity was assessed using trypsin and Nα-benzoyl-DL-arginine-p-nitroanilide (BAPNA) as substrate (Xavier-Filho et al. 1989). Protein extracts (100 µL, 5 mg/mL) were incubated with 15 µL of trypsin (0.1 mg/mL) for 30 min, followed by the addition of 200 µL of BAPNA (1.25 mM). After incubation at 37 °C for 30 min, reactions were stopped with 150 µL of 30% acetic acid, and absorbance was recorded at 405 nm.

### Chitinases

Chitinolytic activity was quantified using a colorimetric assay based on the release of N-acetyl-D-glucosamine (NAG) from colloidal chitin, as described by Oliveira et al. (2020). Briefly, 250 µL of colloidal chitin were incubated with 250 µL of protein extracts (5 mg/mL) at 37 °C for 1 h. Reactions were terminated by boiling for 5 min, cooled on ice, and centrifuged at 10,000 g for 20 min at 10 °C.

To convert chitin oligomers into NAG, 10 µL of β-glucuronidase were added to 300 µL of each supernatant and incubated at 37 °C for 1 h, followed by a second boiling step (5 min). For colorimetric detection, 310 µL of the hydrolysate were mixed with 190 µL of sodium acetate buffer (50 mM, pH 5.2) and 100 µL of potassium tetraborate (0.6 M), heated at 98 °C for 5 min, cooled, and reacted with 1 mL of p-dimethylaminobenzaldehyde (DMAB) reagent at 37 °C for 20 min. Absorbance was measured at 585 nm, and chitinase activity was expressed as nanokatal per microgram of protein (nkat/µgP).

### Osmotins

Osmotins were investigated by enzyme-linked immunosorbent assay (ELISA) and dot blot analysis, as described by Freitas et al. (2015). For ELISA, 150 µL of serial dilutions of protein samples (latex proteins, 1 mg/mL; callus proteins, 5 mg/mL) were applied to 96-well microplates and incubated for 16 h at 4 °C. Plates were washed with PBS containing 0.05% Tween-20, blocked with bovine serum albumin (BSA; 10 mg/mL) for 1 h at 37 °C, and incubated with anti-*C. procera* osmotin (CpOsm) primary antibody (1:5,000) for 2 h at 37 °C. After washing, plates were incubated with alkaline phosphatase-conjugated anti-rabbit IgG (1:10,000) for 2 h at 37 °C. Reactions were developed using p-nitrophenyl phosphate (1 mg/mL) for 30 min in the dark, stopped with 50 µL of 4 N NaOH, and absorbance was measured at 405 nm.

For dot blot analysis, 10 µL of each protein sample (latex proteins, 1 mg/mL; callus proteins, 5 mg/mL) were applied to nitrocellulose membranes and air-dried for 10 min. Membranes were blocked with PBS containing 5% (w/v) nonfat dry milk for 2 h at 25 °C and incubated with anti-*C. procera* osmotin (CpOsm) antibody (1:5,000) for 2 h. After washing, membranes were incubated with alkaline phosphatase-conjugated anti-rabbit IgG (1:10,000) for 2 h. Immunoreactive signals were visualized using BCIP/NBT substrate until the appearance of purple spots.

### Peptidases

Total proteolytic activity was determined using azocasein as substrate (Freitas et al. 2010). Protein samples (100 μL, 1 mg/mL) were pre-activated with 1 mM dithiothreitol (DTT) and incubated with 1% (w/v) azocasein (200 μL). After 60 min, reactions were stopped by the addition of 10% (w/v) trichloroacetic acid (TCA). After centrifugation, supernatants were mixed with 2 M NaOH, and absorbance was measured at 420 nm.

Cysteine peptidase activity was evaluated using Nα-benzoyl-DL-arginine-β-naphthylamide (BANA) as substrate (Abe et al. 1992). Protein samples (100 μL, 1 mg/mL) were incubated with 40 μL of 3 mM dithiothreitol (DTT) and 2 mM EDTA for 10 min at 37 °C. Subsequently, 200 μL of BANA solution (1 mM) were added to the reaction mixture and incubated for an additional 30 min at 37 °C. The reaction was stopped by adding 500 μL of 2% HCl in ethanol followed by 500 μL of 0.06% 4-dimethylaminocinnamaldehyde (DMACA). Absorbance was measured at 540 nm.

Serine peptidase activity was evaluated using Nα-benzoyl-DL-arginine-p-nitroanilide (BAPNA) as substrate (Gomes et al. 2005). In this assay, 100 μL of protein samples (1 mg/mL) were mixed with 200 μL of BAPNA solution (1.25 mM) and incubated for 30 min at 37 °C. The reaction was terminated by adding 150 μL of 30% acetic acid, and absorbance was measured at 405 nm after 10 min.

Peptidase activity was further analyzed by zymography on 12% polyacrylamide gels containing 0.1% gelatin (Freitas et al. 2010). After electrophoresis, gels were washed in 2.5% Triton X-100 for 30 min, incubated in sodium acetate buffer (50 mM, pH 5.0) for 3 h at 37 °C, stained with Coomassie Brilliant Blue R-250, and destained to visualize proteolytic bands. Peptidase detection was also performed by ELISA using anti-*C. procera* peptidase antibodies, as previously described.

To investigate the potential contribution of cysteine peptidases to the antifungal activity, 20 mg of protein samples were dissolved in 10 mL of distilled water containing 10 mM iodoacetamide (IAA), a specific inhibitor of cysteine peptidases. The samples were incubated for 30 min at 25 °C and subsequently dialyzed against distilled water for 48 h using a dialysis membrane with a molecular weight cut-off of 14 kDa (Sigma-Aldrich, D9527) to remove residual IAA (Souza et al. 2011). The dialyzed samples were then lyophilized and used to determine residual cysteine peptidase activity using azocasein as the substrate and to evaluate their antifungal activity.

### Detection of latex cysteine peptidase and osmotin in callus

Total RNA from latex (1 mL) and *C. procera* callus tissues (100 mg) was isolated using the RNeasy Plant Mini Kit (Qiagen), according to the manufacturer’s instructions. Complementary DNA (cDNA) synthesis was performed using the ImProm-II™ Reverse Transcriptase kit (Promega) following the recommended protocol. For RT-PCR assays, the elongation factor 1 alpha gene (EF1α) was used as an internal reference (Souza et al. 2017). Specific primers designed for EF1α, *C. procera* osmotin (CpOsm), and *C. procera* peptidase (CpPep) are listed in Table 1.

**Table 1:** Forward (F) and reverse (R) primers sequences for *EF1α*, *CpOsm*, and *CpPep*. EF1α, Elongation Factor 1 alpha; CpOsm, *Calotropis procera* osmotin; CpPep, *C. procera* peptidase

Primer	Sequence (5′ → 3′)
EF1α F	AGRTTYGAGAARGARGCTGC
EF1α R	CCACGCTTSAGATCCTTVAC
CpOsm F	CCGGCCACNTTYACNATHCGNAACAAYTGYCC
CpOsm R	CCGGGRCARAANAYAACYCTRTARTTDGT
CpPep F	CCTATGGATACTTCGTTAAGT
CpPep R	GATGCTGATATGTCCTTCGTC

Amplification reactions were carried out in a Mastercycler realplex 4S thermocycler (Eppendorf®) using the GoTaq DNA Polymerase kit (Promega), following the manufacturer’s guidelines. The PCR program consisted of an initial denaturation step at 95 °C for 3 min, followed by 33 cycles for osmotin amplification, 37 cycles for peptidase amplification, and 24 cycles for EF1α amplification. Each cycle included denaturation at 94 °C, primer annealing at 57.5 °C, and extension at 72 °C for 1 min each. The reactions were completed with a final extension step at 72 °C for 4 min.

### Statistical analysis

All in vitro antifungal and enzymatic activity assays were conducted using three independent biological replicates, each comprising four technical replicates. Data were analyzed using GraphPad Prism version 6.0. Statistical significance was assessed by one-way analysis of variance (ANOVA), followed by Tukey’s post hoc test, with *P*< 0.05 considered statistically significant.

## Results

### Morphogenic responses and callus induction in *C. grandiflora* explants

**Fig. 1:**
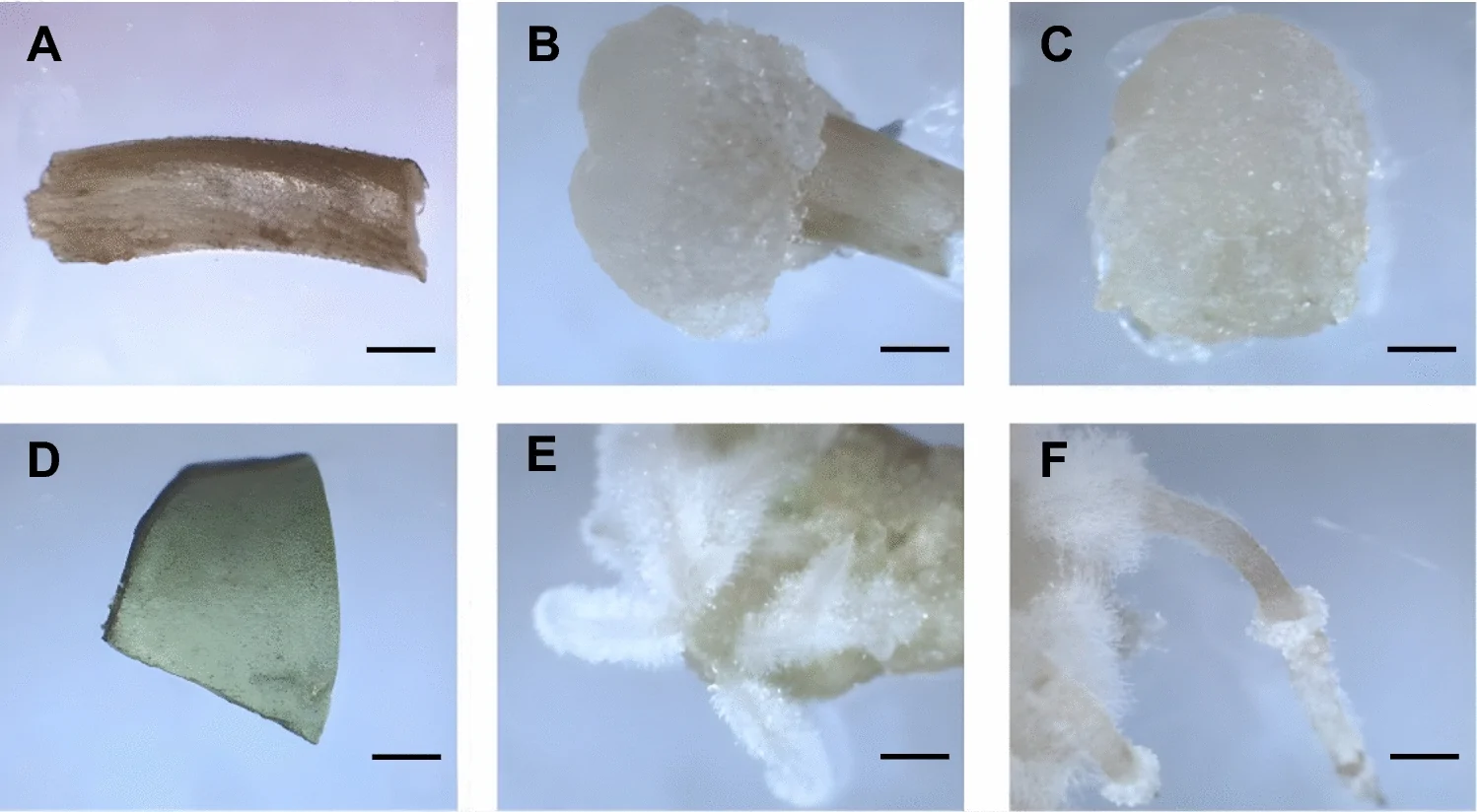
Morphogenic responses of explants of *Cryptostegia grandiflora* cultured on MS medium supplemented with naphthaleneacetic acid and kinetin. **A** Hypocotyl explant prior to inoculation. **B, C** Development of partially friable and yellowish callus after 20 and 35 days of culture, respectively. **D** Cotyledon explant prior to inoculation. **E, F** Adventitious root formation from cotyledon explants, with limited callus proliferation restricted to the explant surface, after 20 and 35 days of culture, respectively. Cultures were maintained in darkness at 25 ± 2 °C. Scale bars = 1 mm

MS medium supplemented with 3.0 μM 1-naphthaleneacetic acid and 4.6 μM kinetin efficiently induced morphogenic responses in both explant types of *C. grandiflora*. Hypocotyl segments (100%) showed a strong capacity for callogenesis, producing partially friable yellowish calli that became visible after 20 days of culture and increased in size by 35 days (Fig. 1A–C). These calli exhibited a granular texture and pale-yellow coloration. In contrast, cotyledon explants (100%) displayed a distinct developmental pattern characterized predominantly by adventitious root formation rather than extensive callus proliferation (Fig. 1D–F).

Root primordia emerged from the explant surface after approximately 20 days and developed into well-defined adventitious roots by 35 days, whereas callus formation remained restricted to the cotyledon surface (Fig. 1E, F). Throughout the culture period, calli derived from hypocotyl segments maintained a friable consistency and pale-yellow coloration without visible signs of tissue oxidation or necrosis, indicating that the culture conditions supported sustained explant growth and morphogenic development.

### Histological analysis

**Fig. 2:**
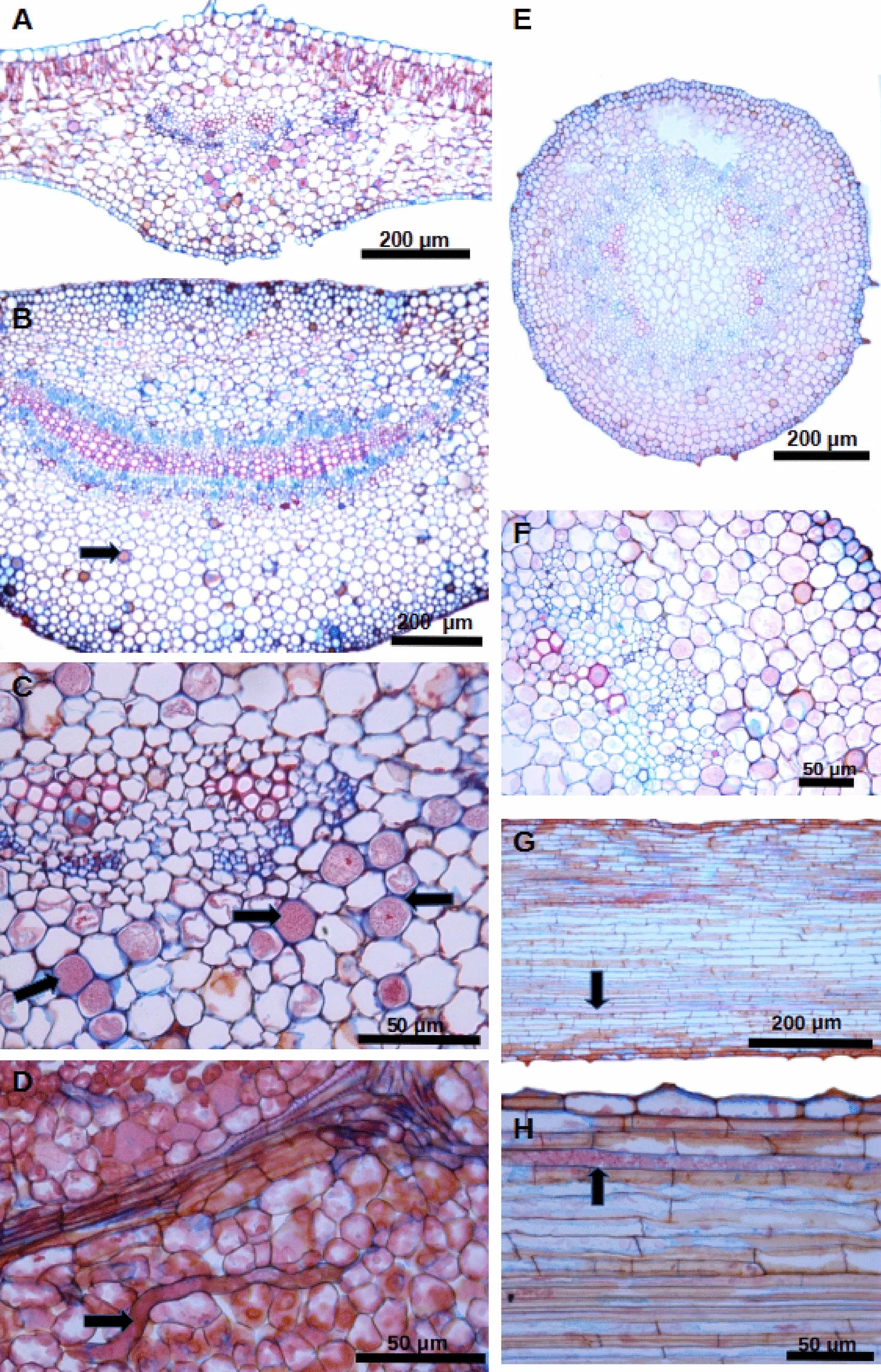
Histological sections of *Cryptostegia grandiflora* cotyledons and hypocotyls showing the distribution of laticifers. **A** Overview of the cotyledon in the region of the midrib. **B** Laticifers located in close association with the vascular bundle. **C** Laticifers observed in the leaf blade near the adaxial epidermis. **D** Paradermal section highlighting a longitudinally oriented laticifer. **E**, **F** Transverse sections of the hypocotyl. **G**, **H** Longitudinal sections of the hypocotyl, highlighting the presence of laticifers. Sections were stained with Astra blue and safranin. Arrows indicate laticifers

Laticifers were detected in both cotyledon and hypocotyl of *C. grandiflora* (Fig. 2). In cotyledons, laticifers were observed in the midrib region as well as mesophyll (Fig. 2A-D). The leaf blade exhibited a uniseriate epidermis followed by two layers of the palisade chlorenchyma and six cell layers of spongy chlorenchyma (Fig. 2A). Within this tissue, laticifers were arranged longitudinally to the leaf veins (Fig. 2D). In the midrib region, they were predominantly associated with the vascular bundles (Fig. 2B, C). In transverse sections, laticifer cells appeared spherical and had pectocellulosic primary cell walls, that were slighly thicker than those of adjacent parenchyma cells. The luminal contents of these cells exhibited a dense appearance consistent with secretory activity (Fig. 2C, D). In hypocotyl tissues, laticifers were consistently observed in the cortical region in both transverse and longitudinal sections (Fig. 2E–H). Transverse sections showed rounded laticifer cells with thick primary cell walls and dense luminal content (Fig. 2E, F) whereas longitudinal and paradermic sections were very elongated (Fig. 2G, H, E, respectively).

**Fig. 3:**
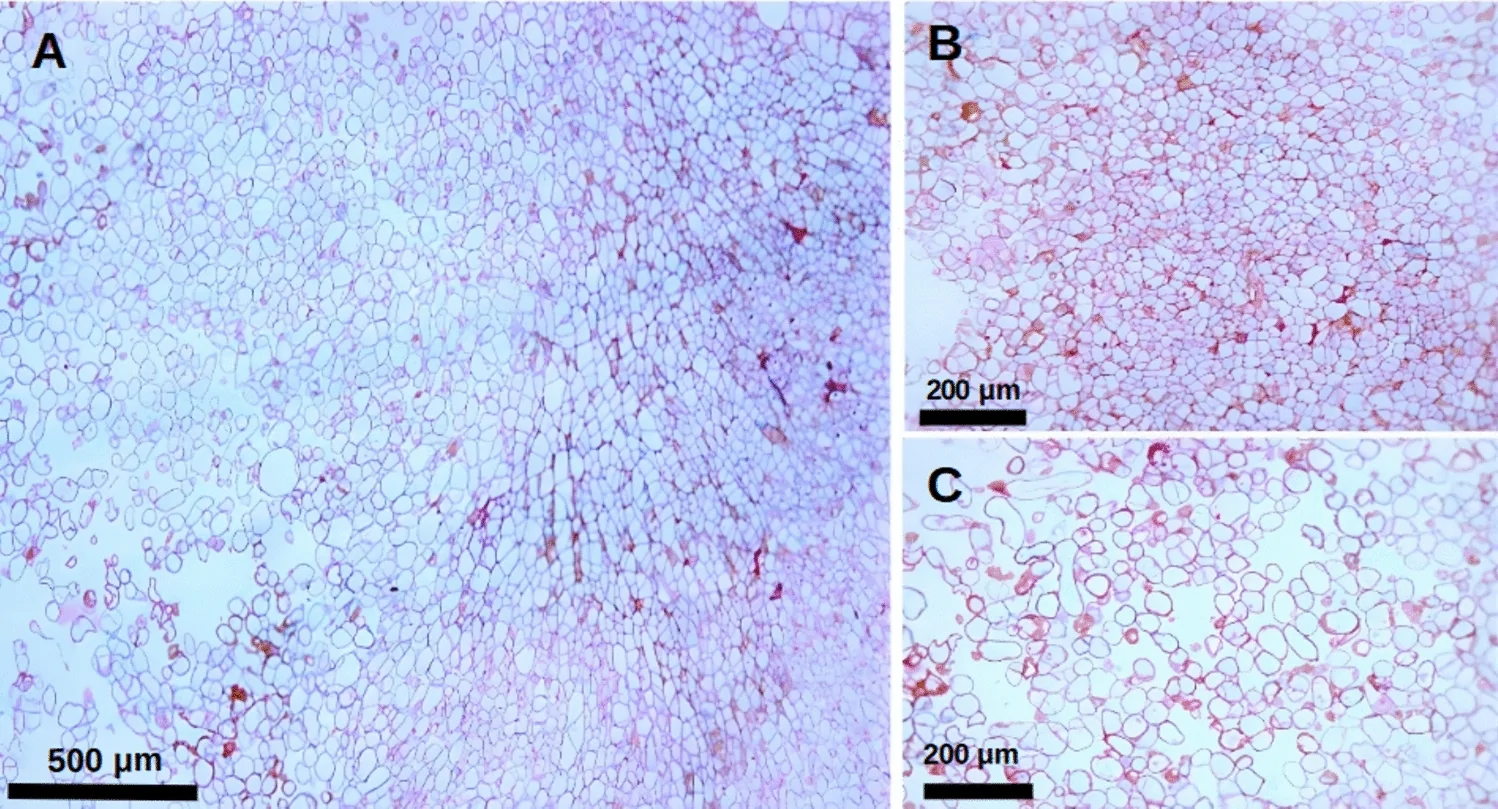
Histological sections of *Cryptostegia grandiflora* callus tissue. **A** Overview of callus structure. **B** Detail showing regions of compact callus. **C** Detail showing regions of friable callus. Sections were stained with Astra blue and safranin

In contrast, laticifers were not detected in *C. grandiflora* calli. Structural analysis revealed two distinct cellular populations within the callus tissue (Fig. 3A). Cells located in the inner regions were densely packed, with reduced cell size, small intercellular spaces, prominent nuclei, and dense cytoplasm, features indicative of intense mitotic activity typical of compact callus tissue (Fig. 3A, B). Conversely, cells near the callus surface possessed primary cell walls, were highly vacuolated, and exhibited large intercellular spaces characteristic of friable callus tissue (Fig. 3C). Consistent with these observations, callus cultures of *C. procera* also lacked detectable laticifers (Teixeira et al. 2011). Based on the absence of laticifers in callus tissues, all subsequent biochemical and biological assays were conducted using protein extracts obtained from calli of *C. grandiflora* and *C. procera*.

### Antifungal activity of callus proteins

**Fig. 4:**
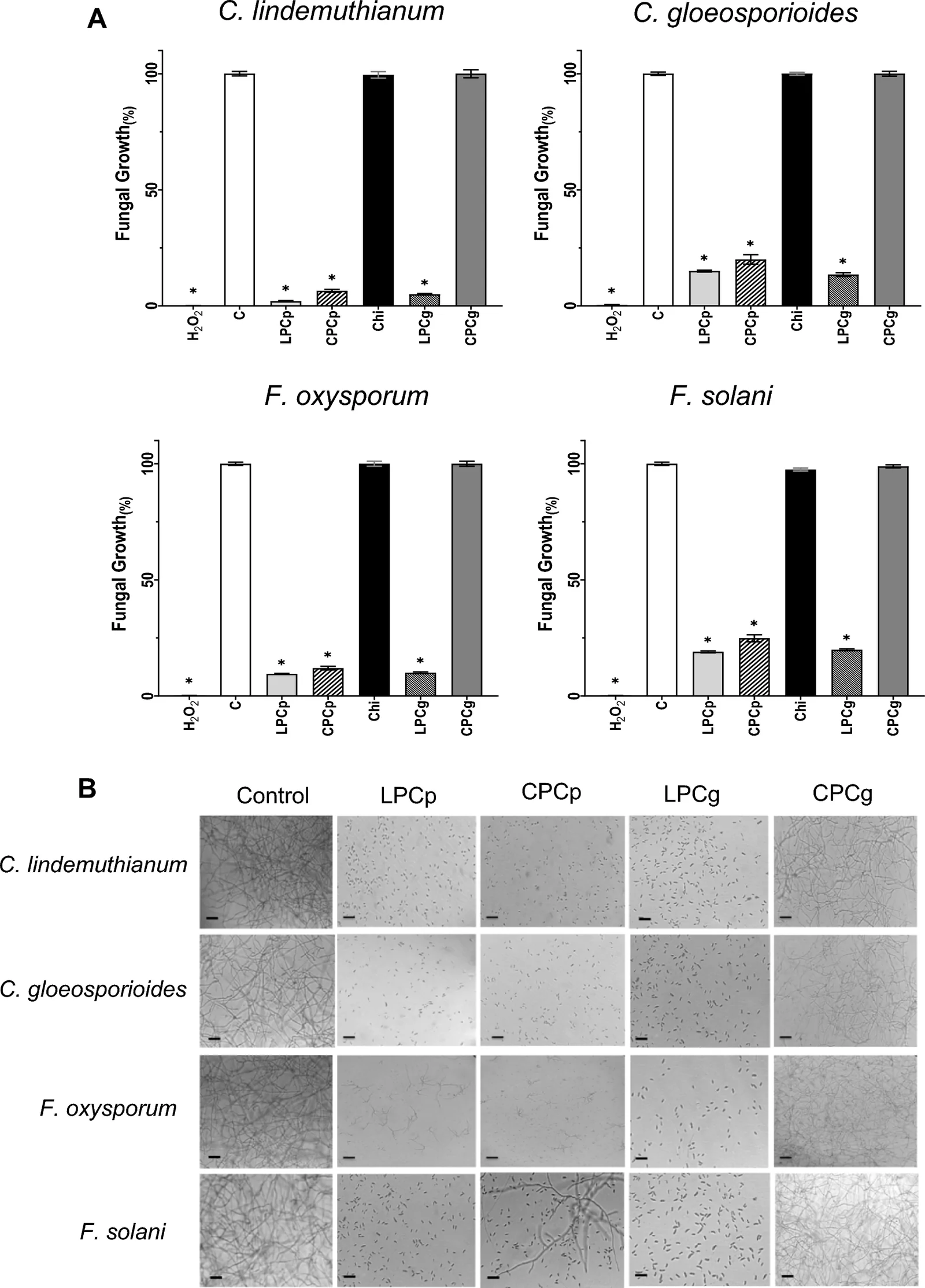
Antifungal activity of latex and callus protein fractions against different phytopathogenic fungi. **A** Inhibition of fungal growth after treatment with protein fractions. Fungal growth is expressed as relative growth (%) compared with control (100%). Data are mean ± SD of three independent biological replicates, each comprising four technical replicates. Asterisks indicate statistically significant differences compared with the control (*P* < 0.05). **B** Representative micrographs of spore germination assays. Treatments include protein fractions from *Calotropis procera* (LPCp and CPCp) and protein fractions from *C. grandiflora* (LPCg and CPCg). Chi, chitinase purified from *C. procera* latex. C, control (sodium acetate buffer pH 5.0). Samples (2.5 mg/mL, final concentration). Bars = 50 µm

The antifungal activity of protein fractions obtained from calli of *C. procera* and *C. grandiflora* was evaluated and compared with that of latex protein fractions from both species. Proteins extracted from *C. procera* calli (CPCp) exhibited strong inhibitory effects on the mycelial growth of all fungal species tested. The inhibition rates were 75.5% for *Fusarium solani*, 88.8% for *F. oxysporum*, 93.7% for *Colletotrichum lindemuthianum*, and 80.2% for *C. gloeosporioides*. In contrast, callus protein extracts from *C. grandiflora* (CPCg) did not inhibit the mycelial growth of any of the fungi evaluated. As expected, latex protein fractions from both species, *C. procera* (LPCp) and *C. grandiflora* (LPCg), displayed antifungal activity against all fungal species tested (Fig. 4A). A similar pattern was observed in the spore germination assays. CPCp inhibited spore germination of all four fungal species, whereas CPCg showed no inhibitory effect. Both latex protein fractions, LPCp and LPCg, also inhibited spore germination of all tested fungi (Fig. 4B).

### Mechanism of action

**Fig. 5:**
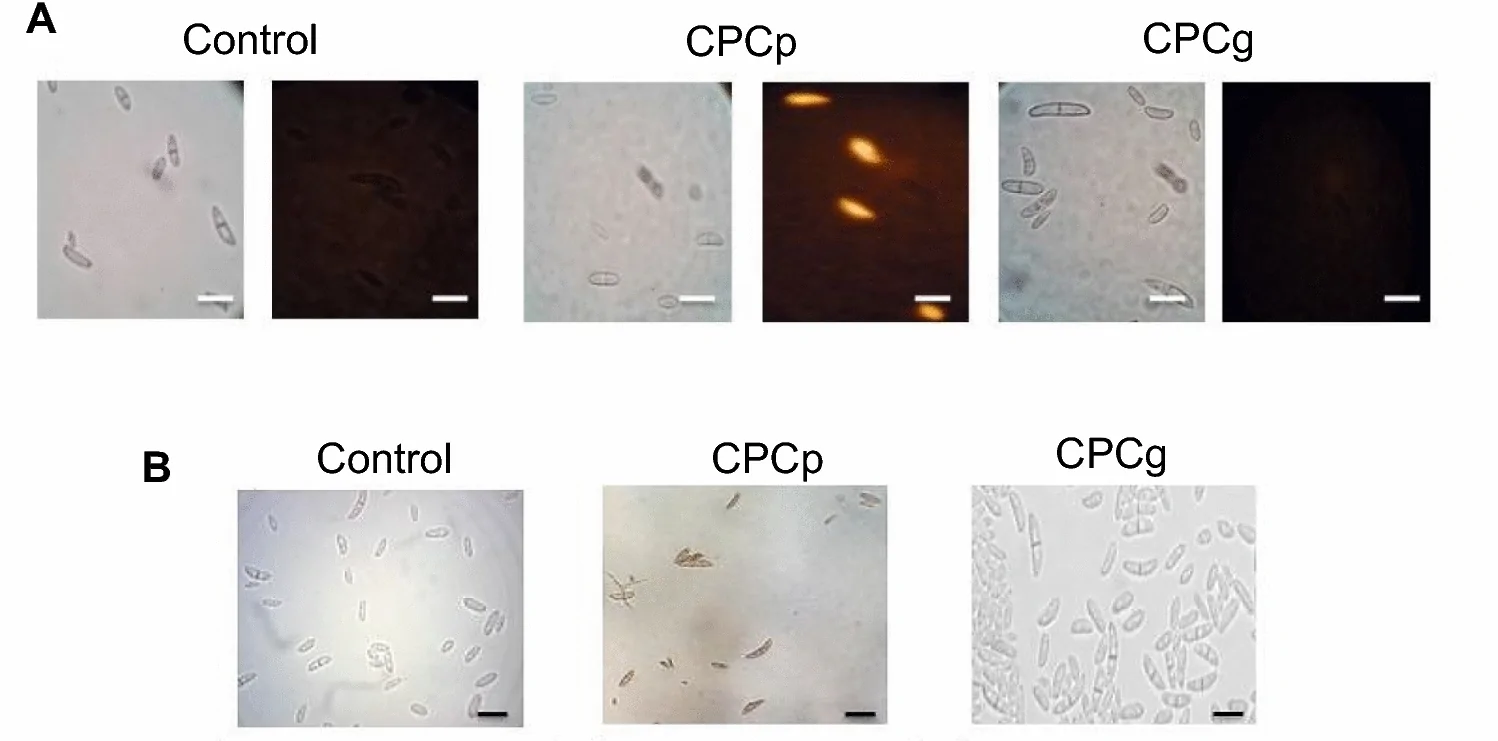
Effects of callus-derived protein fractions on plasma membrane integrity and oxidative stress in *Colletotrichum gloeosporioides* spores. **A** Plasma membrane permeabilization evaluated by propidium iodide staining after treatment with protein fractions. Uptake of propidium iodide indicates loss of membrane integrity. **B** Detection of hydrogen peroxide (H₂O₂) production in fungal spores by 3,3′-diaminobenzidine (DAB) staining, indicating oxidative stress. Control spores were incubated with sodium acetate buffer (pH 5.0). Samples (2.5 mg/mL, final concentration). Scale bars = 10 µm

Latex protein fractions were not included in the following assays because their ability to disrupt fungal plasma membrane integrity and induce oxidative stress has already been demonstrated in previous studies (Souza et al. 2011). Therefore, these experiments focused on determining whether callus-derived protein fractions exhibited similar mechanisms of antifungal action. Based on the antifungal activity profile observed, *C. gloeosporioides* was selected as a representative species for mechanistic analyses. Spores treated with CPCp exhibited clear damage to the plasma membrane, as evidenced by intense fluorescence following propidium iodide staining, indicating increased membrane permeability (Fig. 5A). In contrast, CPCg did not induce detectable membrane damage, as treated spores showed no fluorescence and resembled negative control. The potential induction of oxidative stress was also investigated using DAB staining (Fig. 5B). Spores treated with CPCg did not exhibit reddish-brown precipitates, indicating low levels of hydrogen peroxide accumulation comparable to the negative control. Conversely, spores exposed to CPCp displayed intense reddish-brown staining, indicative of substantial hydrogen peroxide accumulation and oxidative stress associated with the generation of reactive oxygen species (ROS).

### Detection of PR-proteins

**Fig. 6:**
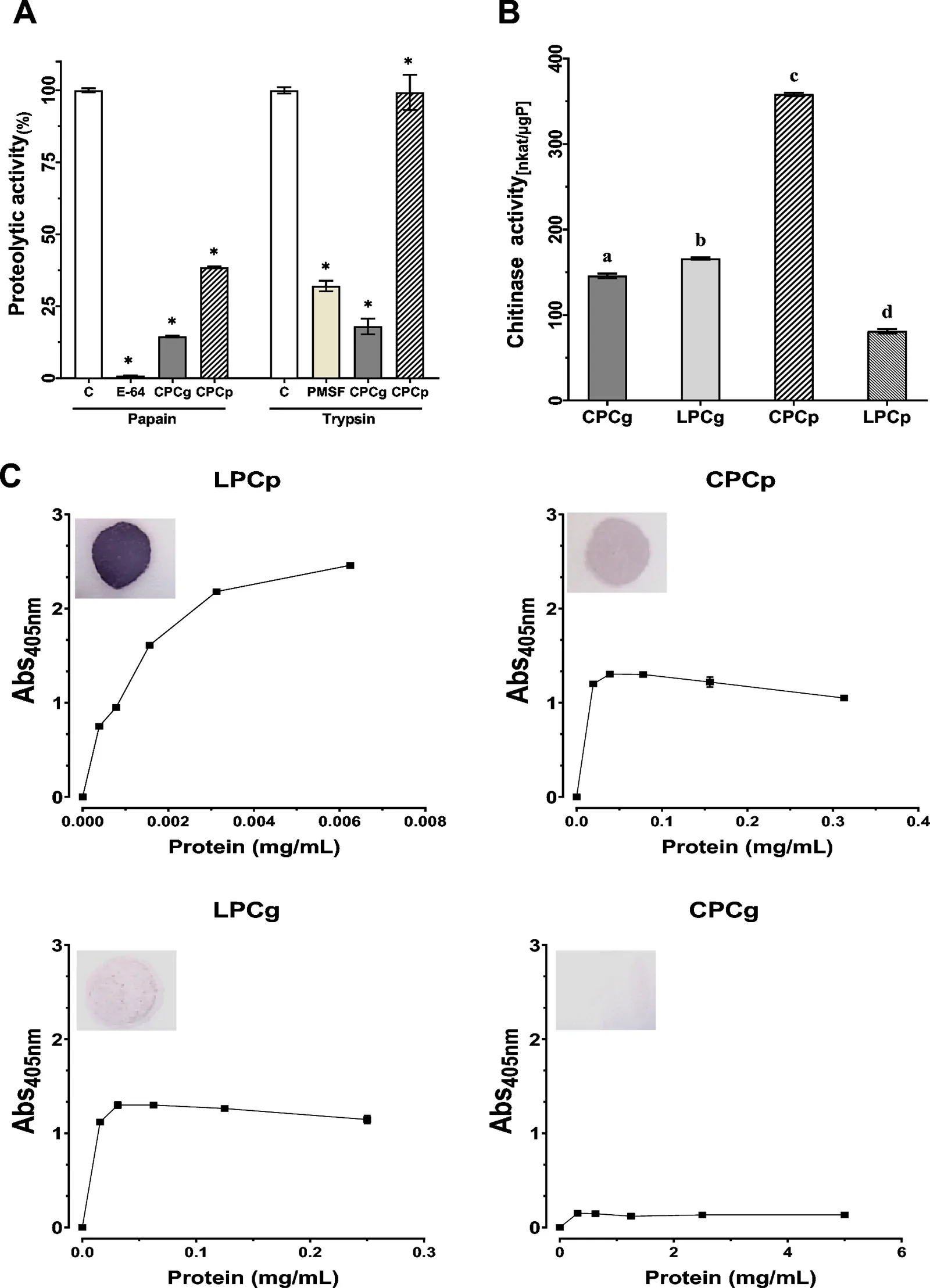
Detection of defense-related proteins in latex and callus protein fractions of *C. procera and C. gradiflora*. **A** Detection of peptidase inhibitors using papain and trypsin inhibition assays. The values represent the relative peptidase inhibitory activity compared with control (C, peptidase). E−64 and PMSF were used as controls. **B** Chitinase activity. **C** Detection of osmotin-like proteins by ELISA using polyclonal antibodies raised against purified osmotin from *Calotropis procera* latex. Insets show the corresponding dot blot assays. Data are mean ± SD of three independent biological replicates, each comprising four technical replicates. Different letters or asterisks indicate statistically significant differences among treatments (*P* < 0.05)

To investigate which defense-related proteins could be associated with the antifungal activity observed, some classes of PR-proteins were analyzed in the callus protein fractions. CPCg inhibited the proteolytic activity of papain and trypsin by 85.3% and 82.1%, respectively, indicating the presence of both cysteine and serine peptidase inhibitors in the callus culture system of *C. grandiflora*. CPCp also inhibited papain activity, with an inhibition rate of 61.6%, but did not affect trypsin activity, suggesting the absence of detectable serine peptidase inhibitors in the *C. procera* callus fraction (Fig. 6A).

Chitinolytic activity was detected in both latex and callus protein fractions of the two species (Fig. 6B). The latex protein fraction of *C. grandiflora* (LPCg) exhibited a chitinolytic activity of 166 nkat/µgP, whereas the corresponding callus fraction (CPCg) showed slightly lower activity (146 nkat/µgP). In contrast, the latex protein fraction of *C. procera* (LPCp) displayed lower activity (81 nkat/µgP), while the callus-derived fraction (CPCp) presented markedly higher activity, reaching 358 nkat/µgP. To further evaluate whether chitinases could contribute to the antifungal activity observed, a purified chitinase fraction obtained from *C. procera* latex was also tested in antifungal assays as reported by Freitas et al. (2016). However, this fraction did not inhibit the growth of any of the four phytopathogenic fungi tested (Fig. 4A).

Osmotins, well-known antifungal proteins previously purified from the latex of *C. procera* (CpOsm) and *C. grandiflora* (CgOsm) (Freitas et al. 2011a, b, 2023), were also investigated. Due to the structural similarity between osmotins from the two species, polyclonal antibodies raised against CpOsm were used to detect osmotins in callus protein fractions. As expected, ELISA assays showed strong recognition of CpOsm in LPCp (Fig. 6C). In contrast, only weak antibody recognition was observed in CPCp, suggesting the presence of an osmotin-related protein that differs from CpOsm. This interpretation is supported by the weak recognition observed in LPCg, which contains an osmotin (CgOsm) structurally similar but not identical to CpOsm (Freitas et al. 2023). No antibody recognition was detected in CPCg, indicating the absence of detectable osmotin-like proteins in this sample. Dot blot assays confirmed these results, showing strong recognition in LPCp, weaker signals in CPCp and LPCg, and no detectable signal in CPCg (Fig. 6C, insets).

**Fig. 7:**
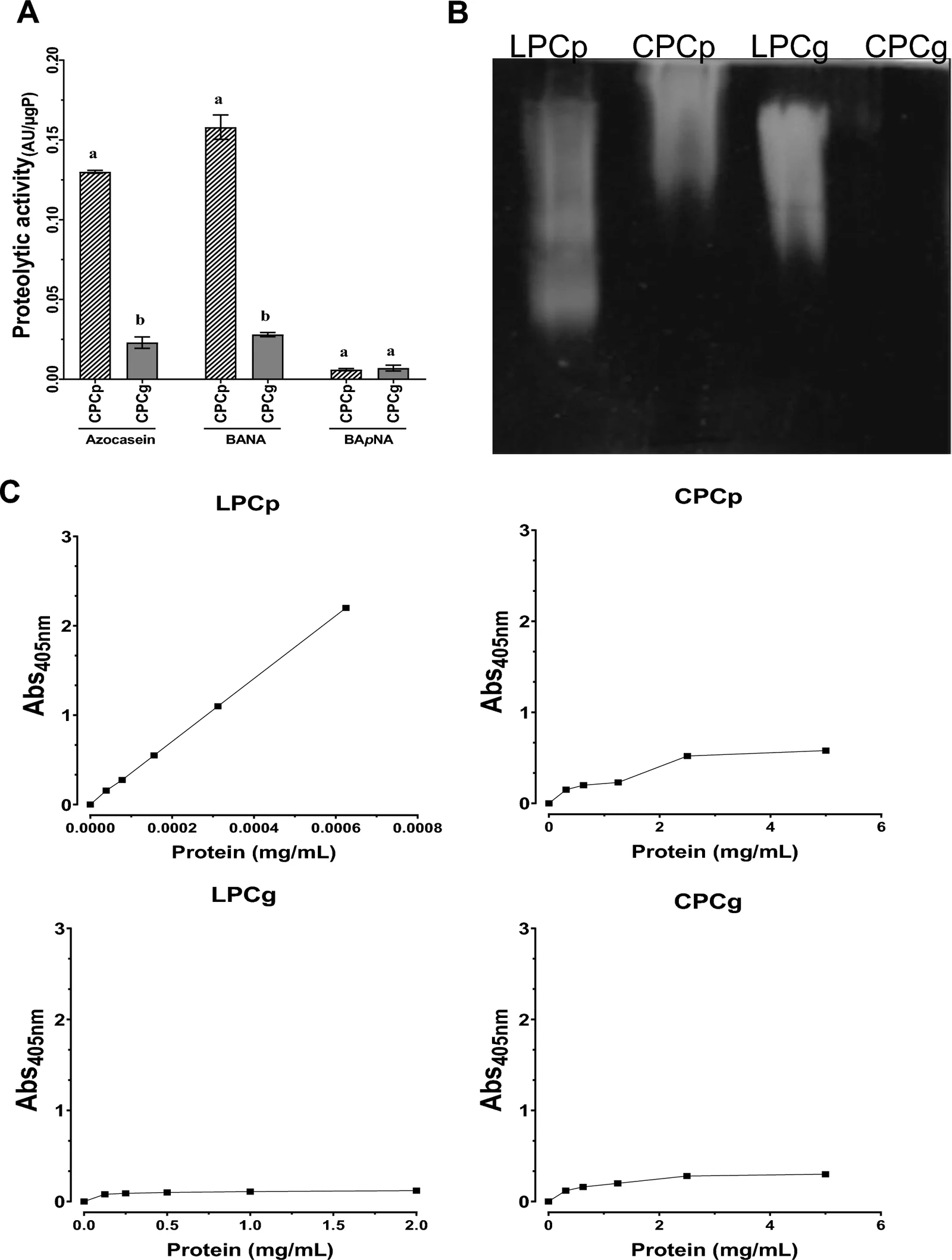
Detection of peptidases in latex and callus protein fractions of *C. procera* and *C. grandiflora. A* Proteolytic activity determined by using different substrates. **B** Gelatin zymography showing peptidase isoforms present in latex and callus fractions. **C** Detection of peptidases by ELISA using polyclonal antibodies raised against purified latex peptidase from *Calotropis procera*. Data are mean ± SD of three independent biological replicates, each comprising four technical replicates. Different letters indicate statistically significant differences among samples (*P* < 0.05)

Because peptidases represent one of the most prominent enzymatic classes in laticiferous fluids and have previously been associated with antifungal activity, the presence of peptidases was further investigated. Proteolytic activity assays revealed consistently higher activity in *C. procera* samples than in *C. grandiflora*, particularly when BANA was used as substrate, suggesting a predominance of cysteine peptidases in CPCp. In contrast, the low activity detected with BAPNA suggests the absence or very low abundance of serine peptidases in the analyzed fractions (Fig. 7A). Zymographic analysis further supported these findings. Clear proteolytic bands were detected in CPCp, confirming the presence of active peptidases in *C. procera* calli, whereas no proteolytic activity was detected in *C. grandiflora* callus extracts. Interestingly, the proteolytic profile observed in CPCp differed from that detected in LPCp, suggesting that the peptidases present in callus tissues are distinct from those found in latex.

To further examine this possibility, ELISA assays were performed using polyclonal antibodies raised against purified *C. procera* latex peptidases. These assays showed only weak antibody recognition of peptidases present in CPCp, supporting the hypothesis that the peptidases expressed in callus tissues differ from those accumulated in latex (Fig. 7C). Consistent with the zymogram results, these antibodies did not recognize peptidases present in LPCg, indicating that the enzymes differ between the two species.

To further evaluate whether cysteine peptidases were responsible for the antifungal activity observed, all protein samples were treated with iodoacetamide (IAA), a specific and irreversible inhibitor of cysteine peptidases. Following IAA treatment, all fractions almost completely lost their proteolytic activity (Supplementary Fig. S1A). Concomitantly, antifungal activity was partially abolished (80–85%) in previously active fractions, including latex and callus proteins of *C. procera* as well as the latex protein fraction of *C. grandiflora* (Supplementary Fig. S1B). These findings support a major contribution of cysteine peptidase activity to the antifungal effects of these protein fractions. However, the residual antifungal activity observed after IAA treatment indicates that cysteine peptidases alone cannot fully account for the antifungal activity, suggesting that other protein components may also contribute to the observed antifungal effects.

**Fig. 8:**
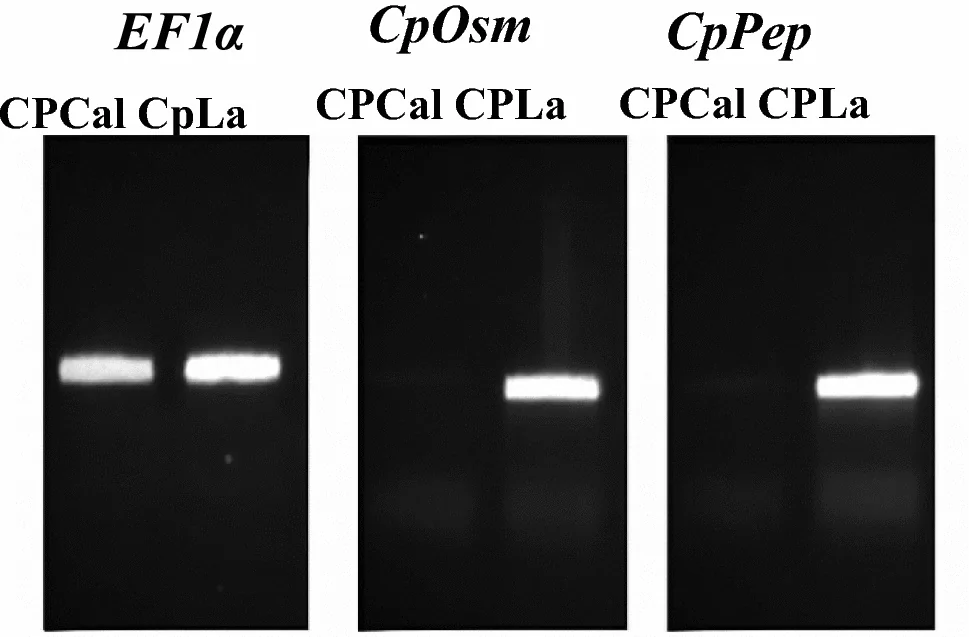
RT-PCR analysis of osmotin (*CpOsm*) and cysteine peptidase (*CpPep*) gene expression in *C. procera* latex and callus tissue. PCR products were separated by 1% agarose gel electrophoresis and visualized by ethidium bromide staining. *EF1α* was used as reference gene. CPCal, *C. procera* callus; CPLa, *C. procera* latex

Finally, to further investigate whether the defense proteins identified in latex were also expressed at the transcript level in non-laticiferous callus tissue, RT-PCR analyses were performed using primers targeting latex cysteine peptidase (CpPep) and osmotin (CpOsm) genes. Transcripts corresponding to the analyzed peptidases and osmotin were detected in latex, whereas no amplification products were detected in the corresponding callus samples under the experimental conditions tested (Fig. 8). This transcriptional pattern was consistent with the biochemical and immunological profiles obtained for the corresponding protein fractions.

## Discussion

Laticifers were detected in cotyledon and hypocotyl tissues of *C. grandiflora*, confirming that this species possesses a well-developed laticifer system distributed across different vegetative tissues. The anatomical organization observed in *C. grandiflora* is consistent with previous descriptions of laticifer distribution in members of the Apocynaceae (Medina et al. 2022). In cotyledon tissues, laticifers were detected both in the midrib region and mesophyll of the leaf blade, frequently associated with vascular bundles and arranged longitudinally within the mesophyll. Similarly, in hypocotyl tissues, laticifers were observed within the cortical region and appeared as elongated tubular structures extending longitudinally through the tissue. A comparable anatomical pattern has been reported for *C. procera*, another laticiferous species of the Apocynaceae (Teixeira et al. 2011). Such anatomical organization is consistent with the defensive role commonly attributed to laticifers. The widespread distribution of these cells across different tissues enables the rapid release of latex upon mechanical injury, generating both a physical and chemical barrier against herbivores and microbial pathogens (Ramos et al. 2020).

In contrast, laticifers were not detected in callus cultures derived from *C. grandiflora* explants. Instead, callus tissues consisted of non-organized cellular masses lacking differentiated secretory structures. The absence of laticifer differentiation in callus tissues likely reflects the highly specialized signaling events required for the formation of these secretory cells (Demarco et al. 2006). Similar limitations in laticifer differentiation under in vitro culture conditions have been reported in other laticiferous species, suggesting that specific time course physiological alteration and tissue organization may be required for laticifer formation (Suri and Ramawat 1995, 1996).

Latex has been widely reported to exhibit antifungal activity, largely attributed to the presence of defense-related proteins such as peptidases, chitinases, osmotins, and other PR-proteins (Ramos et al. 2019; Barbosa et al. 2020). Consistent with these reports, latex protein fractions from both *C. procera* and *C. grandiflora* displayed strong antifungal activity against all fungal species tested in the present study. Interestingly, callus-derived proteins from *C. procera* also exhibited significant antifungal activity, whereas callus proteins from *C. grandiflora* showed no inhibitory effects against the same fungal species. These results indicate that dedifferentiated tissues may also produce antifungal proteins, although this capacity appears to be species-dependent (Mhaskar et al. 2010; Alrayes and Shatnawi 2018).

Mechanistic assays further revealed that the antifungal activity observed in protein fractions from *C. procera* callus was associated with plasma membrane permeabilization and induction of oxidative stress in fungal spores. Both responses have been reported among the cellular effects associated with the activity of plant-derived antifungal proteins and peptides, which may affect fungal membranes, intracellular targets, or multiple cellular processes simultaneously (Chiu et al. 2022; Zou et al. 2023). However, these observations should not be interpreted as direct evidence of the sequence of events underlying the antifungal activity. Membrane permeabilization may represent a primary response to protein–membrane interactions, but it can also occur as a downstream consequence of cellular damage and loss of viability. Similarly, ROS accumulation may reflect oxidative stress directly elicited by antifungal proteins or represent a secondary response of fungal cells to cellular injury and an attempt to restore redox homeostasis. Indeed, the mechanisms described for plant antifungal proteins are frequently complex and involve multiple cellular targets and responses rather than a single defined pathway (Manzanares et al. 2024; Liu et al. 2026).

In contrast, protein fractions obtained from *C. grandiflora* callus, which did not display antifungal activity, were unable to induce membrane damage or reactive oxygen species accumulation in fungal cells. Comparative biochemical analyses of the different protein fractions provide further insight into this difference. The protein fraction derived from *C. procera* callus, which exhibited antifungal activity, contained chitinases, peptidase inhibitors, peptidases, and osmotin-related proteins. Conversely, the protein fraction from *C. grandiflora* callus, which lacked antifungal activity, contained peptidase inhibitors and chitinases but did not exhibit detectable proteolytic activity or osmotins. Importantly, both calli were generated under the same culture conditions and from the same type of explant, allowing their antifungal and protein profiles to be compared under standardized conditions. This comparative profile suggests that chitinases and peptidase inhibitors alone are insufficient to account for the antifungal phenotype, whereas the additional occurrence of proteolytic activity and osmotin-related proteins in *C. procera* callus may contribute to its antifungal properties. However, these findings establish an association rather than a causal relationship between these protein components and antifungal activity. Future studies involving the purification of callus-derived peptidases and osmotin-related proteins, followed by direct assessment of their antifungal activities, would help determine whether these proteins contribute directly to the antifungal phenotype observed in *C. procera* callus.

Proteolytic enzymes are among the most abundant proteins in plant latex and have increasingly been associated with plant defense against phytopathogens (Freitas et al. 2024). In particular, cysteine peptidases have been shown to inhibit fungal growth by damaging fungal cells and interfering with essential cellular processes (Molik et al. 2025). For example, cysteine peptidases purified from *C. procera* latex have been demonstrated to exert antifungal activity against phytopathogenic fungi through mechanisms involving plasma membrane permeabilization, morphological alterations, leakage of cellular contents, and induction of reactive oxygen species in fungal spores (Freitas et al. 2020). Similar effects have also been reported for a cysteine peptidase purified from the latex of *C. grandiflora*, which inhibits fungal spore germination through membrane permeabilization (Ramos et al. 2014). These previously described mechanisms are consistent with the membrane damage and oxidative stress responses observed in the present study. The contribution of cysteine peptidases to the antifungal activity of the protein fractions was further investigated by treatment with iodoacetamide (IAA), an irreversible inhibitor of cysteine peptidases. Following IAA treatment, the proteolytic activity of the analyzed fractions was almost completely abolished, whereas their antifungal activity was markedly reduced but not completely eliminated (Supplementary Fig. S1). This differential effect provides evidence that cysteine peptidase activity makes a substantial contribution to the antifungal activity of the protein fractions, while also indicating that proteolysis alone cannot fully account for the observed antifungal effect. Importantly, because the experiments were performed with complex protein fractions rather than purified enzymes, the possibility that IAA affected other proteins containing reactive sulfhydryl groups cannot be completely excluded. Nevertheless, the samples were extensively dialyzed against distilled water following IAA treatment to remove residual inhibitor before the antifungal assays, thereby minimizing the possibility that free IAA itself contributed to the reduction in fungal growth. Moreover, the persistence of antifungal activity after treatment, despite the near-complete loss of proteolytic activity, indicates that antifungal components other than IAA-sensitive cysteine peptidases remained functionally active (Souza et al. 2011).

Osmotins represent another important class of antifungal proteins detected in laticifer fluids. These proteins belong to the PR-5 family, also known as thaumatin-like proteins (TLPs), and have been widely reported as potent antifungal agents in plants (Ullah et al. 2018). Osmotin purified from *C. procera* latex (CpOsm) has been shown to inhibit both spore germination and mycelial growth of several phytopathogenic fungi, including *Fusarium solani*, *Neurospora* sp., and *Colletotrichum gloeosporioides*, at relatively low inhibitory concentrations (Freitas et al. 2011a). Structural studies of CpOsm demonstrated that this protein is highly stable and capable of binding to negatively charged membrane phospholipids, promoting membrane permeabilization and leakage of intracellular contents (Ramos et al. 2015). In vitro assays have shown that CpOsm induces the uptake of propidium iodide by fungal spores and hyphae, indicating disruption of plasma membrane integrity (Freitas et al. 2011b). Similar membrane-disrupting effects have also been observed for osmotin purified from *C. grandiflora* latex (CgOsm), in which treated fungal spores exhibit severe structural damage and leakage of cytoplasmic material, suggesting the formation of pores in the fungal membrane (Freitas et al. 2023). Osmotins are also known to trigger oxidative imbalance and cellular stress responses in fungal cells, which may culminate in membrane destabilization and growth inhibition (Manghwar and Hussain 2022). Together with cysteine peptidases, osmotins therefore appear to represent key components of the biochemical defense system present in laticifer fluids, acting through complementary mechanisms that combine enzymatic degradation, oxidative stress, and direct disruption of fungal cellular integrity.

Although chitinases are generally considered important components of plant defense due to their ability to hydrolyze chitin in fungal cell walls, their antifungal effects can vary considerably depending on the fungal species (Khan et al. 2024). Studies on chitinases purified from *C. procera* latex have shown that these enzymes exhibit only limited inhibitory effects on fungal growth but display strong insecticidal activity (Freitas et al. 2016). The experimental results obtained in this study further support this interpretation. A purified chitinase fraction obtained from *C. procera* latex did not inhibit the growth of any of the four phytopathogenic fungi tested, indicating that chitinases alone are not sufficient to explain the antifungal activity observed in the latex-derived fractions analyzed here.

Similarly, peptidase inhibitors have been associated with plant defense against insects rather than with direct antifungal activity (Singh et al. 2023). These proteins act primarily by inhibiting digestive peptidases released in the insect gut, thereby impairing nutrient assimilation and ultimately affecting insect metabolism, growth, and survival (Mohammad and Alzweiri 2022). Previous studies on *C. procera* latex proteins have shown that these inhibitors can significantly affect the development of insect pests, reinforcing the role of latex proteins in plant resistance to herbivory (Ramos et al. 2010). In another study, a protein fraction from *C. procera* latex enriched in peptidase inhibitors and devoid of detectable proteolytic activity, obtained following thermal denaturation, did not show antifungal activity (Souza et al. 2011). In the context of the present study, the detection of peptidase inhibitors in both callus and latex fractions that lacked antifungal activity further supports the view that these proteins are not primary determinants of antifungal defense in these species. Instead, their biological role appears to be more closely associated with anti-herbivore defense. This functional specialization is consistent with the multifunctional nature of latex, which acts as a complex defensive secretion capable of targeting different biological threats through distinct biochemical strategies (Agrawal and Konno 2009).

An additional and particularly relevant finding of the present study is that the defense proteins detected in callus tissues differ from those typically found in latex. Although both latex and callus extracts from *C. procera* exhibited antifungal activity, some lines of evidence indicate that the proteins responsible for this activity differ between these two tissues. Zymographic analyses revealed distinct proteolytic profiles in latex and callus fractions, suggesting the presence of different peptidase isoforms. Immunological assays using antibodies raised against latex peptidase and osmotin also showed only weak recognition of callus proteins, indicating limited structural similarity between the proteins present in these tissues. Gene expression analyses further supported this conclusion, as primers specific for the latex cysteine peptidase and osmotin genes previously characterized in *C. procera* failed to detect expression of these genes in callus tissues.

## Conclusion

This study provides new insights into the defensive systems operating in latex plants. Our results suggest that callus-derived proteins from *C. procera* retain antifungal activity associated with the presence of peptidases and osmotins, whereas callus proteins from *C. grandiflora* lack such activity despite containing other PR-related proteins, including chitinases and peptidase inhibitors. These findings propose that peptidases and osmotins are the major determinants of the antifungal activity observed in laticifer fluids, reinforcing their central role in the chemical defense system of Apocynaceae species. In addition, the results reveal that the defense protein isoforms expressed in callus tissues differ from those typically accumulated in latex, suggesting that the biosynthesis of key laticifer proteins is closely associated with cellular differentiation and the development of laticifer cells. Collectively, these findings expand current knowledge on the biological roles of laticifer proteins and highlight the existence of tissue-specific regulation of distinct protein isoforms. Moreover, this study opens new perspectives for future investigations aimed at understanding the molecular mechanisms controlling the gene expression of specific defense proteins as well as their isoforms, particularly those responsible to express peptidase and osmotin genes in latex but not in other plant tissues such as the callus tissue generated under in vitro culture conditions.

## Supplementary Information

Below is the link to the electronic supplementary material.

Supplementary file1 (DOCX 36 KB)

## Data Availability

Data will be made available on request.

## Abbreviations

CPCg: Callus protein fraction of *C. grandiflora*
CPCp: Callus protein fraction of *C. procera*
LPCg: Latex protein fraction of *C. grandiflora*
LPCp: Latex protein fraction of *C. procera*
PR: Pathogenesis-related
