# Supplementary material for: Isoforms of cysteine peptidases and osmotins instead of chitinases and peptidase inhibitors drive antifungal activity but occur as distinct isoforms in latex and callus tissues of Calotropis procera
Source: Planta. 2026 Oct 11;264(5):165. doi: 10.1007/s00425-026-05198-2 (PMC13653838; doi:10.1007/s00425-026-05198-2)
Supplement: Supplementary file 1 [file 425_2026_5198_MOESM1_ESM.docx]

**(B)**

**(A)**

**Supplementary Fig. 1.** **Effect of iodoacetamide (IAA) on the proteolytic activity and antifungal activity of protein fractions from *C. procera* and *C. grandiflora*.** **(A)** Proteolytic activity was determined using azocasein as the substrate at pH 5.0. Samples were incubated with IAA and subsequently dialyzed against distilled water, as described in the Materials and Methods. The untreated fraction was considered to represent 100% activity. **(B)** Antifungal activity against *C. gloeosporioides*. The negative control (sodium acetate buffer, pH 5.0) was considered to represent 100% of fungal growth and was used as the reference for comparison with the other protein fractions. All samples were dissolved in sodium acetate buffer (pH 5.0) at 2.5 mg/mL (final concentration). The CPCg fraction was not included because it exhibited neither detectable proteolytic nor antifungal activity.
